# In Vitro Bioassay-Guided Identification of Anticancer Properties from *Moringa oleifera* Lam. Leaf against the MDA-MB-231 Cell Line

**DOI:** 10.3390/ph13120464

**Published:** 2020-12-15

**Authors:** Prapakorn Wisitpongpun, Nungruthai Suphrom, Pachuen Potup, Nitra Nuengchamnong, Philip C. Calder, Kanchana Usuwanthim

**Affiliations:** 1 Cellular and Molecular Immunology Research Unit (CMIRU), Faculty of Allied Health Sciences, Naresuan University, Phitsanulok 65000, Thailand; prapakornw59@nu.ac.th (P.W.); pachuenp@nu.ac.th (P.P.); 2 Department of Chemistry, Faculty of Science and Center of Excellence for Innovation in Chemistry, Naresuan University, Phitsanulok 65000, Thailand; nungruthais@nu.ac.th; 3 Science Laboratory Centre, Faculty of Science, Naresuan University, Phitsanulok 65000, Thailand; Nitran@nu.ac.th; 4 School of Human Development and Health, Faculty of Medicine, University of Southampton, Southampton SO16 6YD, UK; pcc@southampton.ac.uk

**Keywords:** *Moringa oleifera*, triple negative breast cancer, MDA-MB-231, oleamide, 7-octenoic acid, 1-phenyl-2-pentanol, LC-ESI-QTOF-MS/MS

## Abstract

*Moringa oleifera* Lam. (MO) is a medicinal plant distributed across the Middle East, Asia, and Africa. MO has been used in the traditional treatment of various diseases including cancer. This study aimed to perform bioassay-guided fractionation and identification of bioactive compounds from MO leaf against MDA-MB-231 breast cancer cells. MO leaf was sequentially extracted with hexane, ethyl acetate (EtOAc), and ethanol. The most effective extract was subjected to fractionation. MO extract and its derived fractions were continuously screened for anti-cancer activities. The strongest fraction was selected for re-fractionation and identification of bioactive compounds using LC-ESI-QTOF-MS/MS analysis. The best anticancer activities were related to the fraction no. 7-derived crude EtOAc extract. This fraction significantly reduced cell viability and clonogenic growth and increased cells apoptosis. Moreover, sub-fraction no. 7.7-derived fraction no. 7 was selected for the identification of bioactive compounds. There were 10 candidate compounds tentatively identified by LC-ESI-QTOF-MS. Three of identified compounds (7-octenoic acid, oleamide, and 1-phenyl-2-pentanol) showed anticancer activities by inducing cell cycle arrest and triggering apoptosis through suppressed Bcl-2 expression which subsequently promotes activation of caspase 3, indicators for the apoptosis pathway. This study identified 10 candidate compounds that may have potential in the field of anticancer substances.

## 1. Introduction

Breast cancer is the most frequently diagnosed cancer in women and is one of the leading causes of cancer death for women. Worldwide, over 1.3 million cases of invasive breast cancer are diagnosed and more than 450,000 women die from breast cancer annually [1,2]. Breast cancer is a heterogeneous disease with distinct pathological entities and therefore needs diverse therapeutic interventions. Approximately 10–20% of invasive breast cancers are triple-negative breast cancer (TNBC) which is defined as the absence of estrogen receptor, progesterone receptor, and human epidermal growth factor receptor (HER) 2 [3]. TNBC is associated with poor prognosis, and the survival after metastasis is worse compared to other subtypes [3,4]. Unfortunately, due to lack of targeted therapy, radiotherapy and chemotherapy remain the only recommended option for TNBC [3,5,6]. Therefore, it is urgent to develop new alternative therapies for TNBC.

*Moringa oleifera* Lam. (MO) is a highly valued medicinal plant native to India and now distributed widely across the Middle East, Africa, and Asia, including Thailand. It belongs to the family Moringaceae and is commonly referred to as the “Drumstick tree” [7,8,9]. All parts of the MO possess medicinal properties, and the leaf has the highest nutritional value [10]. MO leaves (MOL) contain high levels of vitamins C and A, potassium, calcium, iron, and proteins. Additionally, the leaves contain phytochemicals like carotenoids, alkaloids, flavonoids, and amino acids, such as cystine, lysine, methionine, and tryptophan [10,11,12]. In vitro and in vivo studies have demonstrated that MOL extract has various biological activities and therapeutic effects, including cardioprotective [13], hypocholesterolaemic [14], neuroprotective [15], anti-inflammatory [16], antioxidant [17,18,19], anti-hypertensive [20,21], antidiabetic [22,23], antibacterial [24,25], immunomodulatory [26,27], and anticancer properties [28,29,30].

With regard to the anticancer properties, MOL extracts have been shown to disrupt the proliferation of different cancer cell lines, for example the hot aqueous MOL extract induced apoptosis in human lung cancer A549 cells by affecting mitochondrial viability in a ROS-dependent manner [29]. It also can induced cell cycle arrest in murine B16F10 melanoma cells by increasing of p53, p21^WAF1/Cip1^ and p27^Kip1^ proteins [30]. The methanolic MOL extract induced apoptosis in human cervical cancer HeLa cells by promoting DNA fragmentation [31]. Moreover, oral administration of cold aqueous MOL extract induced apoptosis of human hepatocellular carcinoma HepG2 cells by affecting the apoptosis-related proteins Bcl-2 and caspase-3 [32]. In breast cancer, most of the previous studies of MOL extracts have used the MCF-7 cell line, a hormone receptor-positive breast cancer model [32,33]. Only one study has investigated the effect of MOL on the TNBC cell line, MDA-MB-231: the authors found that ethanol MOL extract arrested these cells at the G2/M phase and effectively induced apoptosis [32]. However, the underlying mechanism and the bioactive compounds involved have not yet been fully elucidated.

In this study, we investigated the in vitro anticancer effect of MOL extract against MDA-MB-231 cells by bioassay-guided fractionation, and identification of potential bioactive compounds responsible for the observed effects. We found that MOL extracts and derived fractions showed a remarkable anticancer activity with a significant decrease of cell viability, striking reduction of colony formation, and induction of apoptosis and cell cycle arrest at the G2/M phase. Additionally, we tentatively identified 10 bioactive compounds by LC-ESI-QTOF-MS analysis. Three of them (7-octenoic acid, oleamide, and 1-phenyl-2-pentanol) can arrest the cell cycle and induce apoptosis of MDA-MB-231 cells. We also demonstrated the anticancer properties of oleamide on human myelogenous leukemia cell K562 and human squamous cell carcinoma SCC-15.

## 2. Results

### 2.1. Screening for Cytotoxic Effects of Crude Hexane, EtOAc, and EtOH Extracts of MOL

**Figure 1:**
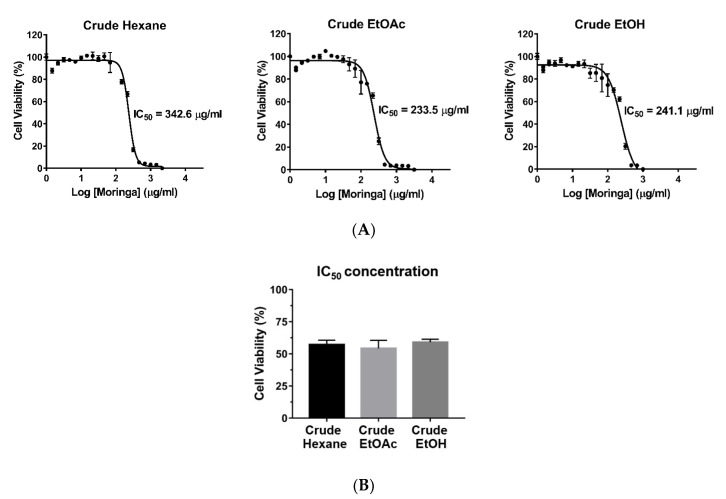
Effects of crude hexane, EtOAc, and EtOH extracts of MOL on the viability of MDA-MB-231 cells. (**A**) Cells were plated into 96-well plates and incubated with each extract for 24 h. IC_50_ values were calculated using GraphPad Prism 6.0 software. Each dot represents mean ± SEM of three independent experiments. (**B**) Cell viability after treatment with each extract at IC_50_ concentrations for 24 h. One-way ANOVA was performed with multiple comparison correction (Dunnett test). Data represent the mean ± SEM of three independent experiments. IC50, the half-maximal inhibitory concentration; EtOAc, ethyl acetate; EtOH, ethanol.

To compare the cytotoxic effects of crude MOL extracts, MDA-MB-231 cells were plated into 96-well plates and incubated with serial concentrations of the crude hexane, crude EtOAc, and crude ethanolic (EtOH) extracts for 24 h. Cell viability was assessed using the MTT assay. Crude EtOAc extract exhibited the lowest IC_50_ value (233.5 μg/mL) followed by crude EtOH extract (241.1 μg/mL), and crude hexane extract (342.6 μg/mL), respectively (Figure 1A,B). The crude EtOAc MOL extract was subjected to further fractionation.

### 2.2. Cytotoxic Effects of EtOAc Extract of MOL and Its Derived Fractions on MDA-MB-231 Cells

**Figure 2:**
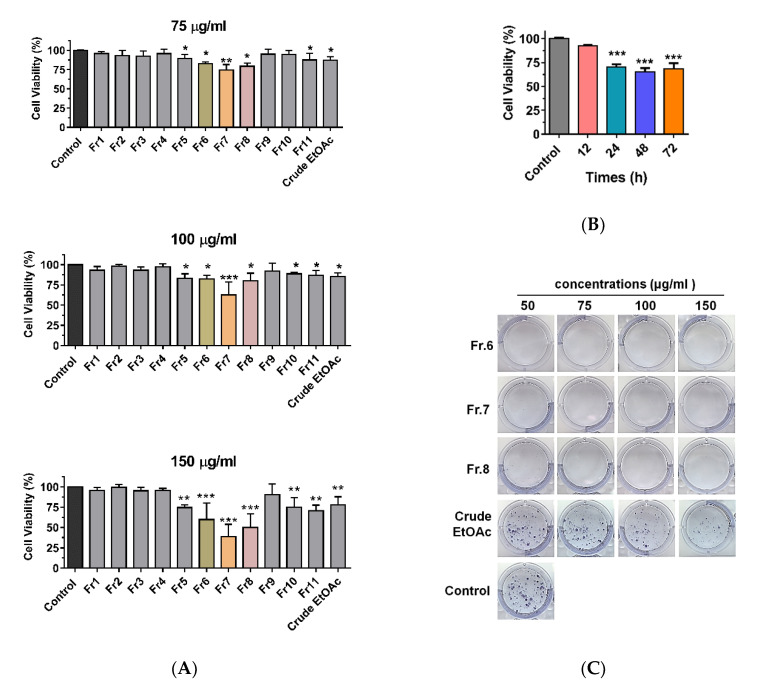
Cytotoxic effect of EtOAc extract and its derived fractions on MDA-MB-231 cells. (**A**) Cells were treated with crude EtOAc extract and fractions no.1-11 at concentrations 70, 100, and 150 µg/mL for 24 h. (**B**) Cells were treated with 100 μg/mL of fraction no. 7 in various times. (**C**) Colony formation assay. One-way ANOVA was performed with multiple comparison correction (Dunnett test). Data represent the mean ± SEM of three independent experiments. (* *p* < 0.05, ** *p* < 0.01, *** *p* < 0.001). EtOAc, ethyl acetate; Fr, fraction.

To examine the inhibitory effects of crude EtOAc extract and its derived fractions, MDA-MB-231 cells were incubated with crude EtOAc extract and fractions no. 1–11 at concentrations of 75, 100, and 150 μg/mL for 24 h. Cell viability was determined using the MTT assay (Figure 2A). Cell viability was significantly decreased after treatment with crude EtOAc extract and fractions no. 5–8 and 10–11. There was a noticeable difference with fractions no. 6–8 showing a significant decrease in cell viability in a dose-dependent manner. The fraction no. 7 showed the strongest cytotoxicity, and thus this fraction was investigated further at different times points (Figure 2B). Cell viability was significantly decreased in time-dependent manner after treatment with 100 μg/mL of fraction no.7. As fractions no. 6–8 exhibited notable effects on viability, we further investigated the impact of crude EtOAc extract and fractions no. 6–8 on the clonogenic growth of MDA-MB-231 cells (Figure 2C and Figure S1). Cells were incubated with crude EtOAc extract or fractions no. 6–8 for 24 h. After incubation, cells were cultured for 14 days in complete medium and then stained with crystal violet to visualize the colonies. Fractions no. 6–8 at concentrations 50–150 µg/mL showed a significant reduction in the number of colonies. These results suggest that the clonogenic growth of MDA-MB-231 cells was completely inhibited by fractions no. 6–8 of MOL extract.

### 2.3. Effect of Crude EtOAc Extract and Fractions No. 6–8 on MDA-MB-231 Cell Apoptosis and Cell-Cycle Arrest

**Figure 3:**
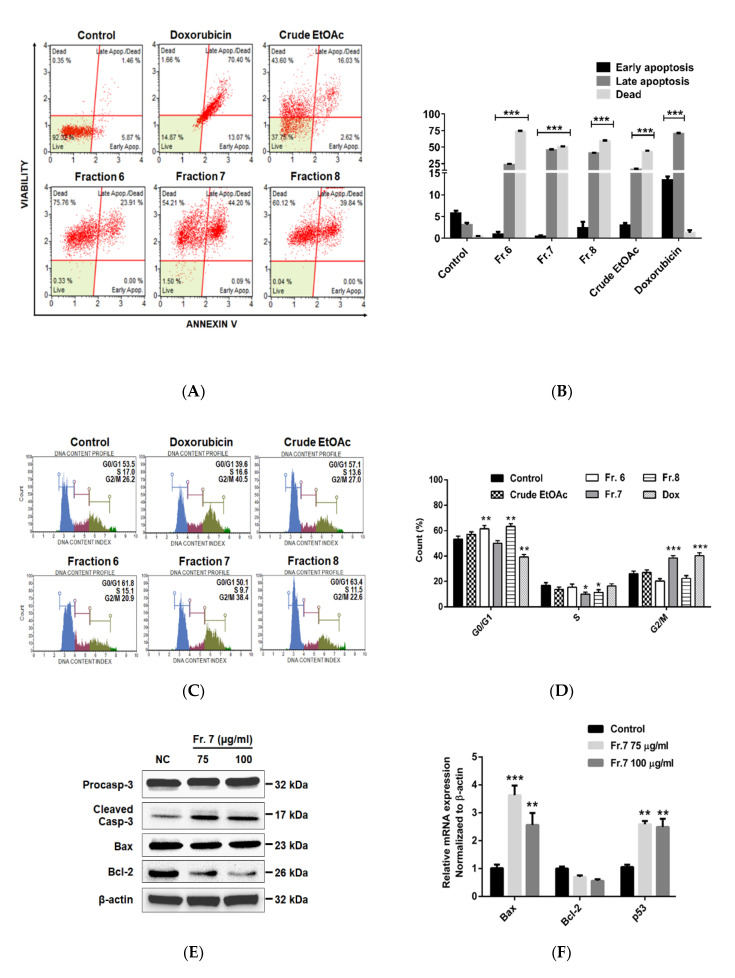
Effect of crude EtOAc extract and fractions no. 6–8 on MDA-MB-231 cell apoptosis and cell-cycle arrest. (**A**–**D**) Cell apoptosis and cell cycle progression analyzed by using Muse cell analyzer. Cells were incubated with crude EtOAc extract or fractions no. 6–8 at 150 µg/mL for 24 h. For untreated control, cells were incubated with complete medium alone. For positive control, cells were incubated with doxorubicin (1.5 µM). (**E**) Western blot analysis. (**F**) mRNA expression by RT-PCR. Cells were incubated with fraction no. 7 for 24 h. One-way ANOVA was performed with multiple comparison correction (Dunnett test). Data represent the mean ± SEM of three independent experiments. (* *p* < 0.05, ** *p* < 0.01, *** *p* < 0.001). EtOAc, ethyl acetate; Fr, fraction; Dox, doxorubicin; NC, negative control; Fr, fraction; β-actin, beta-actin.

To evaluate the role of crude EtOAc extract and fractions no. 6–8 on cell apoptosis and cell-cycle regulation, MDA-MB-231 cells were incubated with crude EtOAc extract and fractions no. 6–8 (150 µg/mL) for 24 h. After incubation, cells were stained with Annexin V/7-AAD and analyzed by a Muse cell analyzer (Merck KGaA, Darmstadt, Germany) (Figure 3A,B and Figure S2). Crude EtOAc extract and fractions no. 6–8 significantly increased the proportion of late apoptotic cells up to 16.0, 23.9, 44.2, and 39.8%, respectively. The proportion of dead cells also increased to 43.6, 75.8, 54.2, and 60.1%, respectively. In addition, the alteration of the cell-cycle distribution was also analyzed (Figure 3C,D and Figure S3). Treatment with fractions no. 6 and 8 resulted in the accumulation of cells at the G0/G1 phase. This varied from 53.5% (control) to 61.8% and 63.4%, respectively. Fraction no. 7 resulted in the accumulation of cells at the G2/M phase (from 26.2% to 38.4%). Therefore, these results suggest that fractions no. 6–8 promoted cell cycle arrest and induced apoptosis of MDA-MB-231 cells.

Since active fraction no. 7 exhibited the strongest anticancer activities, we further investigated the expression of apoptosis-related proteins and mRNA (Figure 3E,F). The expression of certain pro-apoptotic markers, including cleaved caspase 3 protein, Bax mRNA, and p53 mRNA were significantly increased while anti-apoptotic Bcl-2 protein was decreased by fraction no. 7 treatment. These results suggest that the apoptosis induction by MOL fraction no. 7 is through the inhibition of Bcl-2 and the activation p53, Bax, and caspase 3.

### 2.4. Sub-Fractionation of Fraction Bo. 7 and Identification of Compounds by LC-ESI-QTOF-MS/MS

**Figure 4:**
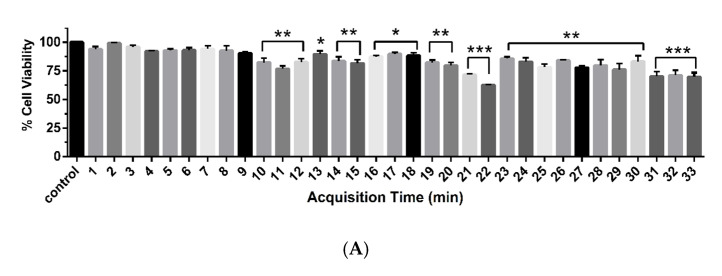

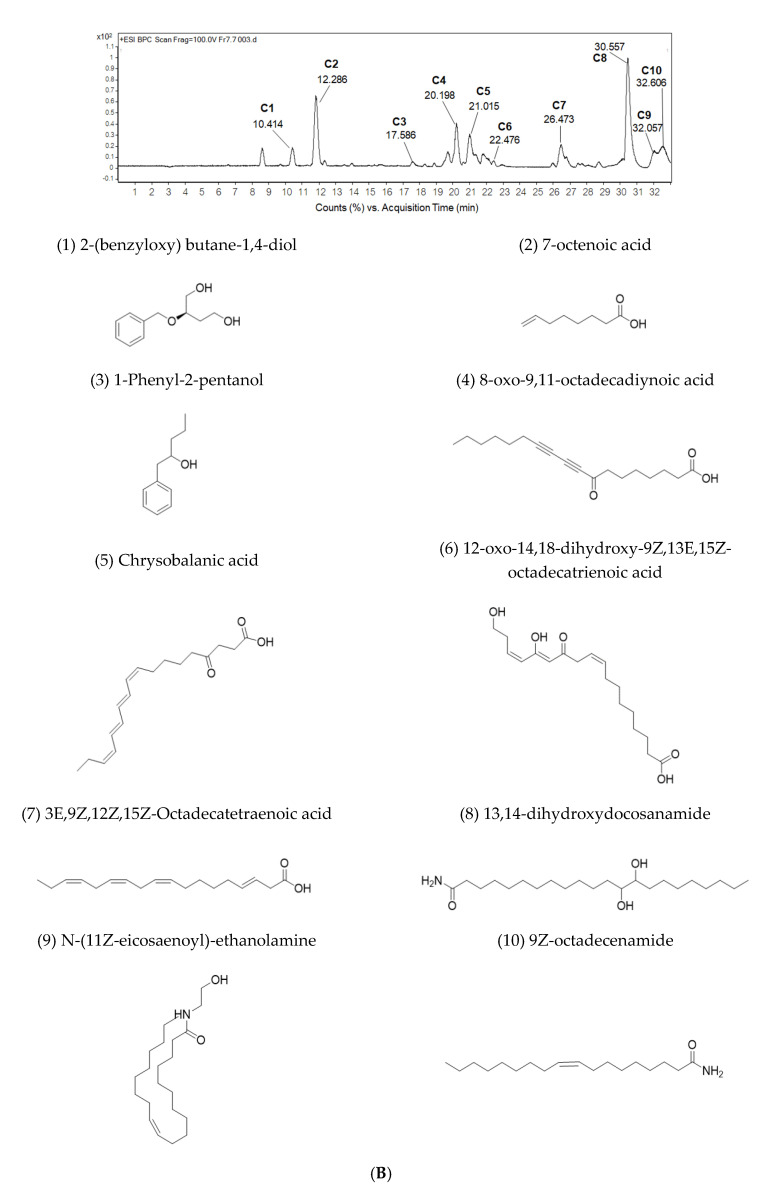
Cytotoxic effect and LC-ESI-QTOF-MS/MS analysis of compounds from sub-fraction no. 7.7. (**A**) Cell viability of MDA-MB-231 cells after treatment with each eluted compound for 24 h. (**B**) LC-ESI-QTOF-MS/MS chromatogram and the identified compounds (C1–C10). One-way ANOVA test was performed with multiple comparison corrections (Dunnett test). Data represent the mean ± SEM of three independent experiments. (* *p* < 0.05, ** *p* < 0.01, *** *p* < 0.001).

To separate the different chemical components in active fraction no. 7, we next re-fractionated fraction no. 7 using silica gel column chromatography. Eight sub-fractions (no. 7.1–7.8) were obtained and their cytotoxicity against MDA-MB-231 cells was assessed using MTT assay (Figure S4). Sub-fraction no. 7.7 strongly reduced the viability of MDA-MB-231 cells compared to the other sub-fractions. Thus, we attempted to tentatively identify the bioactive compounds from sub-fraction no 7.7 using LC-ESI-QTOF-MS/MS. This sub-fraction was injected into an Agilent 1260 Infinity series HPLC system and the constituents were collected in a 96-well plate with 30 s per well until 33 min. In total, ten candidate compounds (C1–C10) were identified. All collected samples were screened for cytotoxicity using the MTT assay and the acquisition times represented active compounds between of 10.414 (C1), 12.286 (C2), 17.586 (C3), 20.198 (C4), 21.015 (C5), 22.476 (C6), 26.473 (C7), 30.557 (C8), 32.057 (C9), and 32.606 (C10) min (Figure 4 and Figure S5). The full tentative identification is listed in Table 1.

### 2.5. The Role of Three-Identified Compounds, 7-Octenoic Acid, Oleamide, and 1-Phenyl-2-Pentanol on MDA-MB-231 Cells Apoptosis and Cell Cycle Progression

**Figure 5:**
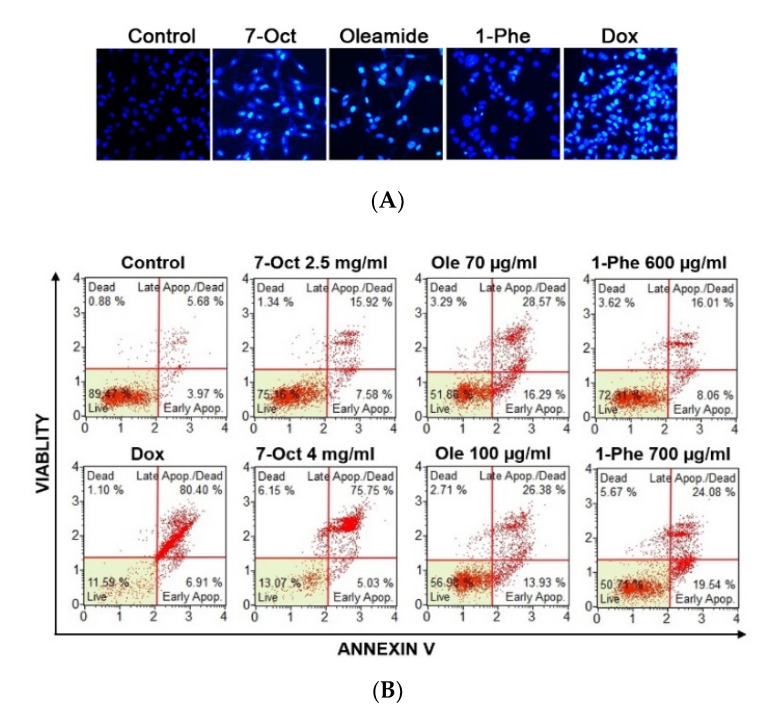

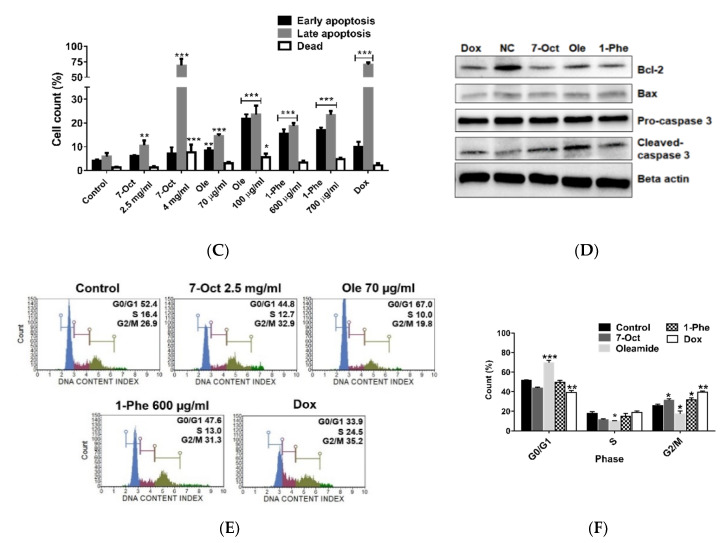
7-octenoic acid (7-Oct), oleamide (Ole), and 1-phenyl-2-pentanol (1-Phe) induced apoptosis and cell cycle arrest in MDA-MB-231 cells. (**A**) Hoechst 33258 staining. (**B**,**C**) Apoptosis analysis using AnnexinV/7-AAD staining. (**D**) Western blot analysis. (**E**,**F**) Cycle cell progression. Cells were incubated with 7-octenoic acid (2.5 mg/mL), oleamide (70 µg/mL), 1-phenyl-2-pentanol (600 µg/mL), or doxorubicin (Dox; 1.5 µM) for 24 h in all experiments. Data represent the mean ± SEM of three independent experiments. (* *p* < 0.05, ** *p* < 0.01, *** *p* < 0.001). 7-Oct, 7-octenoic acid; 1-Phe, 1-Phenyl-2-pentanol; Ole, oleamide; dox, doxorubicin.

Cytotoxicity of compounds on MDA-MB-231 cells was measured by incubating with serial concentrations of 7-octenoic acid, cis-9-octadeceneamide (oleamide), and 1-phenyl-2-pentanol for 24 h (Figure S6). The result showed that cytotoxic dose of oleamide was less than both 1-phenyl-2-pentanol and 7-octenoic acid. To determine the anticancer effect of compounds, we examined the morphologic changes by Hoechst 33342 staining (Figure 5A). An apoptotic morphology was observed after treatments with 7-octenoic acid, oleamide, and 1-phenyl-2-pentanol for 24 h, whereas control cells were round and homogeneously stained. Then apoptosis was assessed by AnnexinV/7-AAD staining with Muse cell analyser (Figure 5B,C). Treatment with 7-octenoic acid significantly increased the proportion of late apoptotic cells. Oleamide and 1-phenyl-2-pentanol significantly increased the proportion of both early and late apoptotic cells. Moreover, the expression of apoptotic-associated proteins; Bcl-2, Bax, pro-caspase 3, and cleaved caspase-3 were measured by Western blot (Figure 5D). We observed an increase of cleaved caspase-3 with the decrease of Bcl-2 in cells treated with the compounds. Additionally, Muse cell analyzer indicated cell cycle arrest (Figure 5E,F). The accumulation of cells in the G0/G1 phase was significantly increased in oleamide treated cells, while 7-octenoic acid and 1-phenyl-2-pentanol resulted in the accumulation of cells at the G2/M phase. These results suggested that the three-identified compounds, 7-octenoic acid, oleamide, and 1-phenyl-2-pentanol, can induce cell cycle arrest and apoptosis of MDA-MB-231 cells.

### 2.6. Bioactive Compounds Suppressed MDA-MB-231 Cell Migration and Induced Apoptosis in Different Cancer Cell Lines

**Figure 6:**
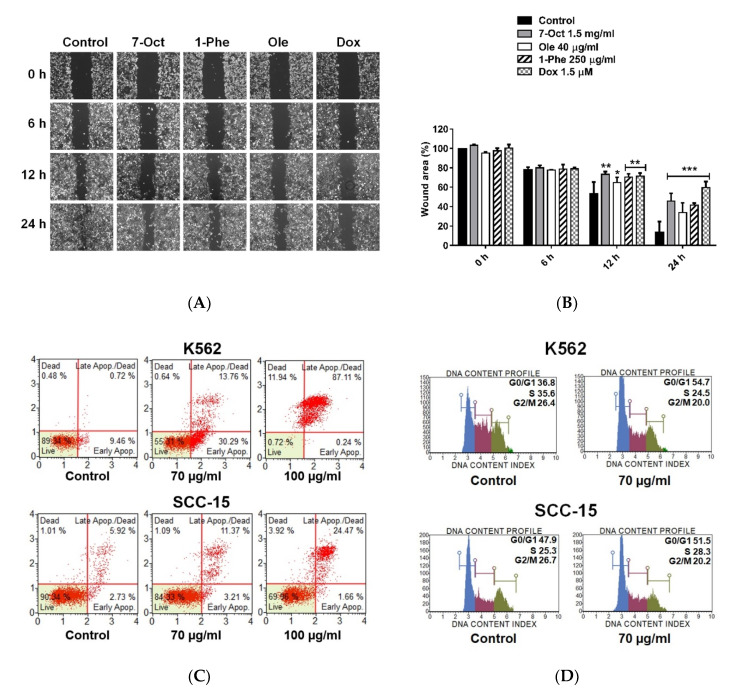
Effect of 7-octenoic acid, oleamide, and 1-phenyl-2-pentanol in vitro cell migration. (**A**) MDA-MB-231 cells were scratch wounded and incubated with compounds, doxorubicin, or complete medium (control). The wound areas were imaged at 0, 6, 12, and 24 h post-scratching. (**B**) Wound area (%) summarized from triplicate data. One-way ANOVA test was performed with multiple comparison corrections (Dunnett test). Data represent the mean ± SEM of three independent experiments. (* *p* < 0.05, ** *p* < 0.01, *** *p* < 0.001). (**C**) Apoptosis and (**D**) cell cycle analysis by Muse cell analyzer. Cells were treatment with compounds for 24 h in all experiments. 7-Oct, 7-octenoic acid; 1-Phe, 1-Phenyl-2-pentanol; Ole, oleamide; dox, doxorubicin.

TNBC is an aggressive form of breast cancer with high metastasis. We determined the effects of the compounds on MDA-MB-231 cell migration using the in vitro scratch assay (Figure 6A,B). The number of migratory cells across the wound regions was significantly decreased after treatment with 7-octenoic acid, oleamide, and 1-phenyl-2-pentanol. This result indicated that 7-octenoic acid, oleamide, and 1-phenyl-2-pentanol may have the ability to inhibit MDA-MB-231 cell migration. As oleamide exhibited notable anticancer effects against MDA-MB-231, we further investigated the effect of oleamide on two different cancer cell lines, including human myelogenous leukemia cell K562 and human squamous cell carcinoma lines SCC-15. Result revealed that treatment with 70 and 100 µg/mL of oleamide increased late apoptosis in both K562 (13.76% and 87.11%) and SCC-15 (11.37% and 24.47%) when compared to the control (Figure 6C). Additionally, cell cycle analysis indicated that oleamide induced cell cycle arrest at G0/G1 phase in both K562 (36.8 to 54.7%) and SCC-15 (47.9 to 51.5%) when compared to the control (Figure 6D). The results suggested that oleamide has potential as an anticancer treatment for MDA-MB-231 TNBC and other cell lines.

### 2.7. Preliminary Prediction of Compound-Targets Interactions with Drug Target Commons

Understanding the binding target of compounds provides important insights into the therapeutic approach. In this study, we preliminary predicted the blinding target of oleamide based on similarity-based approaches and then query the target profiles in drug target commons platform (Table 2) [34]. We found 16 target molecules, including four transmembrane receptors, nine enzymes, and three nuclear proteins. However, the prediction of certain blinding-targets required the combinations of data from multiple platforms such as DrugBank, BindingDB, and Protein Data Bank.

## 3. Discussion

MO is a widely consumed traditional plant in many Asian and South East Asian countries. MO possesses many bioactive compounds with potential health benefits including anti-inflammatory, antioxidant, and anticancer properties [10]. Although much research supports the anticancer effect of MOL, the actual bioactive compounds responsible have not been well characterized. In this study, we explored the anticancer effect, conducted bioassay-guided fractionation, and identified bioactive compounds from MOL extract against the MDA-MB-231 TNBC cell line. We demonstrated that MOL crude EtOAc extract showed the greatest anticancer activity but was non-toxic to primary human macrophage cells (Figure S7). Crude EtOAc extract and its derived fractions were screened for in vitro anticancer activities. Fraction no. 7 was subjected for re-fractionation and finally, the 10 candidate compounds were tentatively identified from the active sub-fraction no. 7.7 using LC-ESI-QTOF-MS. Moreover, we explored the biological activity of three compounds, including 7-octenoic acid, 1-phenyl-2-pentanol, and oleamide against MDA-MB-231, SCC-15, and K562 cell lines.

Previous studies indicated that the tumor suppressor *p53* gene plays an important role in response to DNA damage and other genomic instability. Activation of p53 is crucial in inducing a p53-dependent pathway leading to cell cycle arrest or apoptosis [44]. The up-regulation in *p53* gene expression following MOL fraction no. 7 treatment may explain the cell cycle arrest and apoptosis caused by this fraction (Figure 3). Caspase-3 is a protein that is cleaved and thus activated upon the initiation of apoptosis. Cleaved caspase-3 propagates the apoptotic signal by cleaving downstream executioner caspases at their intersubunit linker [45]. We found that fraction no. 7 treatment of MDA-MB-231 cells resulted in concentration-dependent activation of caspase-3 (Figure 3). The Bcl-2 family of proteins are the central regulators of the mitochondrial cell-intrinsic apoptosis [46]. In contrast, an increase in expression of Bax induces cell death and leads to the elimination of tumor cells [46,47]. Therefore, we measured the expression of Bcl-2 family proteins, and we found that fraction no. 7 treatment resulted in increased expression of Bax and decreased expression of Bcl-2. Collectively, our results reveal that MOL fraction no. 7 up-regulates pro-apoptotic p53, cleaved caspase-3, and Bax, while down-regulating anti-apoptotic Bcl-2 protein levels.

On the other hand, flow cytometry analysis with annexin V and PI staining indicated that MOL fraction no. 7 was not only able to induce apoptosis but also triggered necrotic cell death in MDA-MB-231 cells. This mechanism might be caused by the activation of caspase-3 which triggers cross-talk to necrotic signaling, resulting in secondary necrosis [48,49]. The main features of secondary necrosis are osmotic cell swelling and lysis that lead to leakage of the cell contents; thereby it may cause tissue injury and induction of inflammation and other immune responses if the dying cells are not quickly removed by phagocytes [48,49]. MOL fraction no. 7 induced MDA-MB-231 cell cycle arrest at G2/M phase similar to the study of Al-Asmari et al., that found increased MDA-MB-231 cell accumulation in the G2/M phase after treatment with crude EtOAc extract [50].

Previously, MOL has been described as containing many phenolic compounds that exert anticancer effects including quercetin, kaempferol, isothiocyanate, and gallic acid [12,51]. Moreover, long-chain fatty acids and their derivatives are also considered as anticancer compounds. In this study, based on bioassay-guided fractionation and LC-ESI-QTOF-MS analysis, we identified 10 candidate anticancer compounds (Table 1), including two aromatic compounds (C1 and C3), five fatty acids (C2, C5-C8), two fatty amides (C9 and C10) and a lactone compound (C4). Octadecadienoic acid and oleamide have been previously described in MOL extracts [52,53,54] as seen here. Based on commercially available compounds, we selected three candidate compounds (oleamide, 7-octenoic acid, and 1-phenyl-2-pentanol) to explore cytotoxicity against MDA-MB-231 cells. Oleamide, also known as oleyl amide or 9-octadecenamide, belongs to the class of fatty amides. These are carboxylic acid amide derivatives of fatty acids, that are formed from a fatty acid and an amine. Oleamide was discovered in the cerebrospinal fluid of sleep-deprived cats [55]. It has been observed that oleamide possesses several biological activities especially sleep induction [56] and immunological suppression [57]. Moreover, oleamide has been reported to inhibit gap junction communication on human breast cancer MDA-MB-231 cells [58,59]. It has been reported that 9-octadecenoic acid found about 20.89% in MOL [60]. 7-octenoic acid is a long-chain fatty acid and 1-phenyl-2-pentanol is used as a flavoring agent. There biological activity has not been reported before. In the present study, we demonstrate the anticancer activity of these compounds against breast cancer cells; we found that oleamide exhibits the strongest anticancer activity when compared to the other two compounds. However, of 1-phenyl-2-pentanol and 7-octadecenoic acid may act in combination or synergistically with other compounds to enhance their activity. We present preliminary results of the synergistic effect of identified compounds (Figure S8) after incubation for 24 h. This requires further investigation. Our research provides ten candidate bioactive compounds that might be useful for investigating for anti-cancer activity and future development of treatments for TNBC and other type of cancer. Although we identified the potential compounds by studying their mechanisms of actions, their interaction or combination effect remains unclear. Several previous studies demonstrated that combination therapy can improve the efficacy of the drug in TNBC cell lines [61,62,63]. For example, combination treatment of selumetinib (MEK1/2 inhibitor) with buparlisib (PI3K inhibitor) is synergistic in MDA-MB-231 cells [61]. Therefore, the future investigation of anticancer drugs and active natural product combination with chemotherapy, radiotherapy, and other treatments is important. Additionally, the limitation of this study is performed only MDA-MB-231, TNBC model. It is unclear whether these compounds still show the same efficacy as other TNBC cell lines. Thus, further study should be performed in other TNBC cell lines such as MDA-MB-175, MDA-MB-435, and MDA-MB-436 [64]. However, this study demonstrated that the anticancer efficacy of oleamide with other cancer cell lines, including human myelogenous leukemia cell K562 and human squamous cell carcinoma lines SCC-15, which revealed that oleamide has potential anticancer activity for other cancer types. Recently, patient-derived cancer models have been proposed to gradually replace the cancer cell lines for translational research [65,66,67]. For example, lung cancer patients derived samples were studied the effective drug combination responds [65]. T-cell prolymphocytic leukemia-derived model were successfully used for identification and prediction of drug combination treatment [67]. In the present study, we demonstrate the bioassay-guided fractionation and identification of the potential anticancer compounds from MOL extract against TNBC cell lines MDA-MB-231. There are 10 compounds were tentatively identified by LC-ESI-QTOF-MS analysis. Moreover, also demonstrates the anticancer properties of compounds no. 2 (7-octenoic acid), no. 3 (1-phenyl-2-pentanol), and no. 9 (oleamide) against MDA-MB-231 cell and other cancer lines including SCC-15 and K562. Therefore, further investigation of MOL extract and bioactive compounds on TNBC patient derived samples and patient-derived xenograft models are powerful tool for combination effects and to overcome the resistance mechanisms.

## 4. Materials and Methods

### 4.1. Cell Culture and Chemicals

MDA-MB-231, SCC15, and K562 were obtained from American Type Cell Collection (Manassas, VA, USA). They were cultured in Dulbecco’s modified eagle medium (Gibco, Carlsbad, CA, USA), DMEM/F12 (Gibco, Carlsbad, CA, USA) and RPMI 1640 (Gibco, Carlsbad, CA, USA), respectively. Medium cultured were supplemented with 10% fetal bovine serum (Gibco, Carlsbad, CA, USA) and 1% penicillin/streptomycin (Gibco, Carlsbad, CA, USA). Cells were cultured in a humidified atmosphere with 5% CO_2_ at 37^o^C. Primary antibodies against β-actin, Bax, Bcl-2, caspase-3 were from Santa Cruz (Santa Cruz, CA, USA), and anti-cleaved caspase-3 was from Affinity Biosciences (Cincinnati, OH, USA). Mouse anti-human IgG1 Fc secondary antibody conjugated with horseradish peroxidase (HRP) and 3-(4,5-Dimethylthiazol-2-yl)-2,5-Diphenyltetrazolium Bromide (MTT) were from Thermo Fischer Scientific (Waltham, MA, USA). Oleamide, 7-octenoic acid, and 1-phenyl-2-entanol were from Sigma Aldrich (St. Louis, MO, USA).

### 4.2. Extraction and Fractionation of MOL Extract

MOL dried powder (COA lot no. 5534) from Khaolaor Laboratories Co., Ltd. (Samutprakan, Thailand) was subjected to serial exhaustive extraction. One kilogram of MOL powder was sequentially extracted using hexane, ethyl acetate (EtOAc), and 95% ethanol (EtOH) as extractants. The extraction process was repeated three times with 1 L of each solvent at room temperature (RT). The extracts were filtered through Whatman no. 3 filter paper and the filtrates were concentrated using a rotary evaporator (Heidolph Hei-VAP Value HB/G3B) to produce hexane extract (35.37 g), EtOAc extract (44.78 g), and EtOH extract (66.63 g). The crude EtOAc extract (10 g) was dissolved in hexane-EtOAc (70–30%) and separated by chromatography on a silica gel column; a gradient sequentially formed of hexane-EtOAc (90–10%, 80–20%, 70–30%, 60–40%, 50–50%, 40–60%, 30–70%, 20–80%, 10–90%, and 100% EtOAc), and EtOAc-methanol (MeOH) (90–10%, 80–20%, and 100% MeOH) were used as the mobile phase for elution. All collected fractions were monitored and combined based on the thin layer chromatography (TLC) pattern. Next, the active fraction (fraction no. 7; 135 mg) was dissolved in hexane-EtOAc (30–70%) and re-fractionated by silica gel column chromatography; a gradient sequentially formed of hexane-EtOAc (90–10%, 80–20%, 70–30%, 50–50%, and 100% EtOAc), and EtOAc-MeOH (90–10%) were used as the mobile phase for elution. All sub-fractions (nos. 7.1–7.8) were tested for anticancer activity using the MTT assay. The strongest active sub-fraction (sub-fraction no. 7.7) was then subjected to the identification of bioactive compounds using at-line-LC-ESI-QTOF-MS/MS analysis. All extracts were stored at −20 °C until use.

### 4.3. At-Line-LC-ESI-QTOF-MS/MS Analysis

The active subfraction of MOL extract (20 mg/mL) was prepared and injected into an Agilent 1260 Infinity Series HPLC system (Agilent Technologies Inc., Waldbronn, Germany) coupled to an Agilent 6540 QTOF-MS spectrometer (Agilent Technologies, Inc., Singapore) with an electrospray ionization (ESI) source. For HPLC separation, a mobile phase of 0.1% formic acid in water (*v*/*v*) (A) and 0.1% formic acid in acetonitrile (*v*/*v*) (B) was used with gradient elution from 20% B to 90% B in 30 min, hold on 3 min and post-run for 5 min. The sample separation was performed on a Luna C18 (2) 100°A, 4.6 × 150 mm, 5 μm column (serial no. 728946-40; Phenomenex, CA, USA) at a flow rate of 0.5 mL/min, with the column temperature set at 35 °C. The eluent was split into two flows using a 9:1 ratio. The major part was collected in a 96-well plate with 30 s per well, while the minor part flowed to an ESI-QTOF-MS system. The operating parameters for MS detection were as follows: drying gas (N_2_) flow rate 10.0 L/min; drying gas temperature 350 °C; nebulizer pressure 30 psig; capillary 3500 V; skimmer 65 V; octapole RFV 750 V; and fragmentor voltage 100 V in positive mode. The mass range was set at m/z 100–1200 amu with a 250 ms/spectrum. The mass fragmentation was operated on auto ms/ms mode with three collision energies of 10, 20, and 40 eV, respectively. All acquisition and analysis of data were controlled by Agilent MassHunter Data Acquisition Software B.05.01 and Agilent MassHunter Qualitative Analysis Software B.06.0, respectively. The micro-fractions in a 96-well plate were dried using a sample concentrator (Techne, Staffordshire, UK) and kept at −20 °C before being tested.

### 4.4. Identification of Active Compounds

The samples from the 96 well plate that showed bioactivity were linked to the LC-ESI-QTOF-MS/MS chromatogram by time. The active compounds with MS and MS/MS data were tentatively identified: the mass data were compared with previous reports and using public databases ((the Human Metabolomics Database; http://www.hmdb.ca; accessed on 30 December 2019), Chemspider (http://www.chemspider.com; accessed on 30 December 2019), and Metlin database (https://metlin.scripps.edu; accessed on 30 December 2019)).

### 4.5. Cell Viability Assay

The cytotoxicity of the MOL extract and its derived fractions were determined using the methyl thiazol tetrazolium (MTT) assay. MDA-MB-231 cells were seeded into 96-well plates at a density of 1 × 10^4^ cells/well and treated with MOL extract at concentration 75, 100, and 150 µg/mL for 24 h. MTT salt solution was added and the plate was incubated for 3 h at 37 °C. Then, formazan crystals were dissolved in 100 µl DMSO. The absorbance was measured at 570 nm using an ELISA plate reader (PerkinElmer, Inc., Waltham, MA, USA).

### 4.6. Colony Formation Assay

MDA-MB-231 cells were seeded into 6-well plates at a density of 500 cells/well and incubated for 24 h. Cells were treated with MOL extract or its derived fractions for 24 h and the plates were then cultured for 14 days in complete DMEM. After 14 days, cells were fixed with 10% neutral formalin and stained with 0.5% crystal violet to visualize colonies and photographed.

### 4.7. Apoptosis and Cell Cycle Analysis

Cell apoptosis and cell cycle analysis were examined on the Muse Cell Analyzer (EMD Millipore, Billerica, MA, USA). MDA-MB-231 cells were plated into 24-well plates at the density of 5 × 10^4^ cells/well. Cells were incubated with MOL extract (150 µg/mL), fractions no. 1–11 (150 µg/mL), 7-octenoic acid (2.5 and 4 mg/mL), oleamide (70 and 100 μg/mL), 1-phenyl-2-pentanol (600 and 700 μg/mL), or doxorubicin (1.5 μM) for 24 h. Cell apoptosis was examined by staining with Muse™ Annexin V and Dead Cell reagent (EMD Millipore, Billerica, MA, USA; cat. no. MCH100105). For cell cycle analysis, cells were fixed in chilled 75% ethanol for 3 h at −20 °C. Then cells were washed with PBS and incubated with Muse™ Cell Cycle Reagent (Millipore, Billerica, MA, USA; cat. no. MCH100106) for 30 min in the dark at RT. Cells were read on the Muse cell analyser.

### 4.8. Cell Migration Assay

Migration of MDA-MB-231 cells was examined by using the in vitro scratch assay. MAD-MB-231 cells (1 × 10^6^) were plated into 6-well plates and cultured for 24 h to form monolayers. The wound area was created by scratching with a SPLScar™ Scratcher. Cells were then incubated with 7-octenoic acid (1.5 mg/mL), oleamide (40 µg/mL), 1-Phenyl-2-pentanol (250 µg/mL), doxorubicin (1.5 µM), or media alone for 24 h. The images were captured at 0, 6, 12, and 24 h using an inverted microscope (Zeiss Microscopy, Oberkochen, Germany). The percentage of wound closure was calculated using ImageJ software (Version 1.52a, Madison, WI, USA).

### 4.9. Hoechst Staining

To assess the effects of compounds on nuclear material, MDA-MB-231 cells were stained with Hoechst 33342. MDA-MB-231 cells were seeded at a density of 7 × 10^5^ cells/well in a 6-well plate containing glass cover slips. Cells were treated with 7-octenoic acid (2.5 mg/mL), oleamide (70 µg/mL), and 1-phenyl-2-pentanol (600 µg/mL) for 24 h. After incubation, cells were washed with PBS, fixed with 4% formaldehyde, and stained with Hoechst 33,342 (4 µg/mL) for 10 min in dark. Cover slides were then mounted with 70% glycerol and images were captured with an inverted microscope using 350 nm excitation and 450 nm emission filters (Zeiss Microscopy, Oberkochen, Germany). A total of three images per treatment were captured at 40× magnification.

### 4.10. Reverse Transcription Quantitative Real-Time PCR (RT-qPCR)

MDA-MB-231 cells (1 × 10^5^) were plated into a 12-well culture plate. Cells were then treated with fraction no. 7 (75 and 100 µg/mL) for 24 h. For control, cells were incubated with culture medium alone. The total RNA was isolated by using Trizol reagent (Invitrogen, Carlsbad, CA, USA) according to the manufacturer’s instructions. First-strand cDNA was synthesized using Tetro cDNA Synthesis Kit (BIOLINE USA Inc, Taunton, MA, USA). The resulting cDNAs were amplified with different primers (Supplement Table S1) using the SensiFAST™ SYBR ^®^ No-ROX Kit (BIOLINE USA Inc., Taunton, MA, USA). Relative differences in gene expression among groups were determined from the quantification cycle (Cq) values. These values were first normalized to a housekeeping gene (β-actin) in the same sample (ΔCq) and are expressed as the fold-change over control (2^−ΔΔCq^). Real-time fluorescence detection was performed using a CFX96 Touch Real-Time PCR Detection System (Bio-Rad, Hercules, CA, USA).

### 4.11. Western Blot Analysis

MDA-MB-231 cells (7 × 10^5^) were plated into 6-well culture plates. Cells were incubated with fraction no. 7 (75 and 100 μg/mL), 7-octenoic acid (2.5 and 4 mg/mL), oleamide (70 and 100 μg/mL), 1-phenyl-2-pentanol (600 and 700 μg/mL), or doxorubicin (1.5 μM) for 24 h. Cells were lysed in RIPA buffer (Bio Basic Inc.) supplemented with Protease/Phosphatase Inhibitor Cocktail (Sigma Aldrich, St. Louis, MO, USA) for 30 min and then centrifuged at 14,000× *g* for 15 min at 4 °C. Protein concentration was determined by the Bradford assay. Equal amounts of protein samples were heated, separated in 12% SDS-PAGE (150 V, 1 h), and transferred onto nitrocellulose membranes (100 V, 1.30 h). The membranes were washed and blocked with 2% BSA in Tris-buffered saline containing 0.5% Tween 20 (TBST) for 1 h at RT. Then the membranes were probed with primary antibodies against β-actin, Bax, Bcl-2, caspase-3, or cleaved caspase-3 by incubating overnight at 4 ˚C. The membranes were then washed three-time with TBST and incubated with secondary antibodies for 1 h at RT. Protein detection was performed by adding horseradish peroxidase chemiluminescence substrate. The intensity of each band was analyzed using Image Lab Software (Version 5.1, Hercules, CA, USA).

### 4.12. Statistical Analysis

Data are shown as mean ± standard error (SEM) of three independent experiments. For comparisons of more than two groups one-way ANOVA were performed with multiple comparison correction (Dunnett test) using GraphPad Prism 6.0 software. *p*-values < 0.05 were considered statistically significant.

## 5. Conclusions

**Figure 7:**
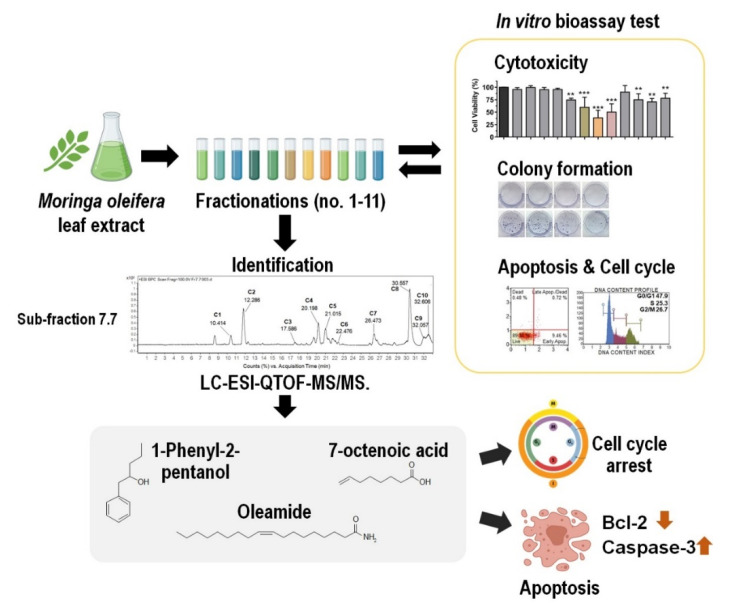
Bioassay-guided fractionation and identification of potential anticancer compounds from MOL extract against TNBC cell lines MDA-MB-231. Crude EtOAc extract was fractionated (no.1–11) and screened for anticancer activities with different in vitro bioassay tests. The strongest fraction was selected for sub-fractionation and identification of bioactive compounds using LC-ESI-QTOF-MS/MS. Three candidate compounds (1-phenyl-2-pentanol, oleamide, and 7-octenoic acid) exhibited anticancer effects through inducing cell cycle arrest. Additionally, suppression of Bcl-2 resulting in the disruption of mitochondrial membrane permeability and subsequent release of apoptogenic factors such as cytochrome c, which activates caspase 3 and finally causes cell apoptosis (** *p* < 0.01, *** *p* < 0.001).

We demonstrate the bioassay-guided fractionation and identification of the potential anticancer compounds from MOL extract against TNBC cell lines MDA-MB-231. Crude EtOAc extract and their fractions were screened for anticancer activity using different bioassay. We tentatively identified 10 compounds from sub-fraction no 7.7 by LC-ESI-QTOF-MS. Moreover, we also investigate the individual mechanisms of actions of three identified compounds, including 7-octenoic acid, oleamide, and 1-phenyl-2-pentanol against TNBC cell lines (Figure 7). Our findings suggest that oleamide has strongest potential as an anticancer treatment by induce apoptosis through suppress Bcl-2 expression and subsequently promote activation of caspase 3. This is the first report concerning MOL extract and oleamide activity against MDA-MB-231 cell line.

## Figures and Tables

**Table 1:** Tentative identification of bioactive compounds identified in sub-fraction 7.7 of MOL extract by LC-ESI-QTOF-MS.

No.	RT (min)	m/z [M + H]+	MS/MS	Tentative Identification	Formula	Error (ppm)
C1	10.414	197.1166	179.1015, 161.0911, 135.1127, 107.0822	2 -(benzyloxy) butane-1,4-diol	C_11_H_16_O_3_	3.15
C2	12.286	143.1057	128.0550, 101.0912, 83.0814, 62.9783, 59.0458, 55.0513	7-octenoic acid	C_8_H_14_O_2_	6.68
C3	17.586	165.1272	147.1157, 95.0482	1-Phenyl-2-pentanol	C_11_H_16_O	1.16
C4	20.198	291.1955	273.1828	8-oxo-9,11-octadecadiynoic acid	C_18_H_26_O_3_	−0.79
C5	21.015	291.1958	273.1806, 171.1019	4-oxo-octadeca-9Z,11E,13E,15Z-tetraenoic acid, Chrysobalanic acid	C_18_H_26_O_3_	−1.13
C6	22.476	325.2013	291.1925, 233.1518, 137.0949	12-oxo-14,18-dihydroxy-9Z,13E,15Z-octadecatrienoic acid	C_18_H_28_O_5_	−1.08
C7	26.473	277.2148	135.1125, 93.0669, 79.0517	3E,9Z,12Z,15Z-Octadecatetraenoic acid	C_18_H_28_O_2_	5.09
C8	30.557	372.3457	354.3303, 337.3052, 319.2933, 97.0993, 83.0840	13,14-dihydroxydocosanamide	C_22_H_45_NO_3_	2.75
C9	32.057	354.3379	337.3075, 319.2965, 301.2865	N-(11Z-eicosaenoyl)-ethanolamine	C_22_H_43_NO_2_	−3.51
C10	32.606	282.2784	*	9Z-octadecenamide	C_18_H_35_NO	2.63

* data not determined.

**Table 2:** Prediction of oleamide blinding-targets with drug target commons.

Target Preferred Name	Gene Names	Target Class	References
1. GABA-A receptor β3 subunit	*GABRB3*	Ion channel	[35,36]
2. Cannabinoid receptor 1 (CB1)	*CNR1*	GPCR	[36]
3. 5-HT_2A_ receptor	*HTR2A*	GPCR	[37,38]
4. P2Y receptors	*P2RY*	GPCR	[39]
5. Fatty acid amide hydrolase	*FAAH*	Enzyme	[40,41,42]
6. Acyl coenzyme A: cholesterol acyltransferase 1	*Soat1*	Enzyme	[43]
7. Cytochrome p450 2c19	*Cyp2c19*	Enzyme	Pubchem bioassay
8. Cytochrome p450 2c19	*Cyp2c19*	Enzyme	Pubchem bioassay
9. Cytochrome P450 2C9	*Cyp2c9*	Enzyme	Pubchem bioassay
10. Cytochrome P450 2D6	*Cyp2d6*	Enzyme	Pubchem bioassay
11. Cytochrome P450 3A4	*Cyp3a4*	Enzyme	Pubchem bioassay
12. Cytochrome p450 1a2	*Cyp1a2*	Enzyme	Pubchem bioassay
13. Ubiquitin carboxyl-terminal hydrolase 1	*Usp1*	Kinase	Pubchem bioassay
14. NF-kappa-B, p105 subunit	*Nfkb1*	Nuclear receptor	Pubchem bioassay
15. Proto-oncogene c-jun	*Jun*	Nuclear receptor	Pubchem bioassay
16. Cellular tumor antigen p53	*Tp53*	Nuclear receptor	Pubchem bioassay

## Supplementary Materials

The following are available online at https://www.mdpi.com/1424-8247/13/12/464/s1, Figure S1: Colony formation assay, Figure S2: Induction of apoptosis in MDA-MB-231 cells, Figure S3: Effect of MOL extract and its derived fractions on the distribution of MDA-MB-231 cells in the cell cycle, Figure S4: Cell viability of MDA-MB-231 cells after treatment with sub-fractions no.7.1-7.8, Figure S5: Overlays of LC-MS chromatograms of MO sub-fraction no.7.7 with active compounds no.1-10. Figure S6: Cytotoxicity of identified compounds on MDA-MB-231 cells, Figure S7: Cell viability of primary human macrophage after treatment with crude EtOAc extract, Figure S8: Combination effect of compounds on MDA-MB-231 cell viability, Figure S9: Original Images of Blots and Gels, Table S1: Primer sequences used in RT-qPCR assay.
