# Supplementary material for: In Vitro Bioassay-Guided Identification of Anticancer Properties from Moringa oleifera Lam. Leaf against the MDA-MB-231 Cell Line
Source: Pharmaceuticals (Basel). 2020 Dec 15;13(12):464. doi: 10.3390/ph13120464 (PMC7765196; doi:10.3390/ph13120464)
Supplement: Supplementary file 1 [file pharmaceuticals-13-00464-s001.pdf]

Article

In Vitro Bioassay-Guided Identification of Anticancer Properties from *Moringa oleifera* Lam. Leaf against the MDA-MB-231 Cell Line

Prapakorn Wisitpongpun [1], Nungruthai Suphrom [2], Pachuen Potup [1], Nitra Nuengchamnong [3], Philip C. Calder [4] and Kanchana Usuwanthim [1,*]

1 Cellular and Molecular Immunology Research Unit (CMIRU), Faculty of Allied Health Sciences, Naresuan University, Phitsanulok 65000, Thailand; prapakornw59@nu.ac.th (P.W.); pachuenp@nu.ac.th (P.P.)

2 Department of Chemistry, Faculty of Science and Center of Excellence for Innovation in Chemistry, Naresuan University, Phitsanulok 65000, Thailand; nungruthais@nu.ac.th

3 Science Laboratory Centre, Faculty of Science, Naresuan University, Phitsanulok 65000, Thailand; Nitran@nu.ac.th

4 School of Human Development and Health, Faculty of Medicine, University of Southampton, Southampton SO16 6YD, UK; pcc@southampton.ac.uk

* Correspondence: kanchanau@nu.ac.th; Tel.: +66-89-780-3878

Supplementary Table S1. Primer sequences used in RT-qPCR assay.

Primer name	Primer sequence Gene
Bcl-2	Fw: 5' – GATGTGATGCCTCTGCGAAG – 3’<br>Rw: 5' – CTAGCTGATGTCTCTGGAATCT – 3’
Bax	Fw: 5' – GGTTGTCGCCCTTTTCTA – 3’<br>Rw: 5' – CGGAGGAAGTCCAATGTC – 3’
p53	Fw: 5' – GTTCCGAGAGCTGAATGAGG – 3’<br>Rw: 5' – TCTGAGTCAGGCCCTTCTGT – 3’
β-actin	Fw: 5' – AGAAAATCTGGCACCACACC – 3’<br>Rw: 5' – CCATCTCTTGCTCGAAGTCC – 3’

Supplementary Figures

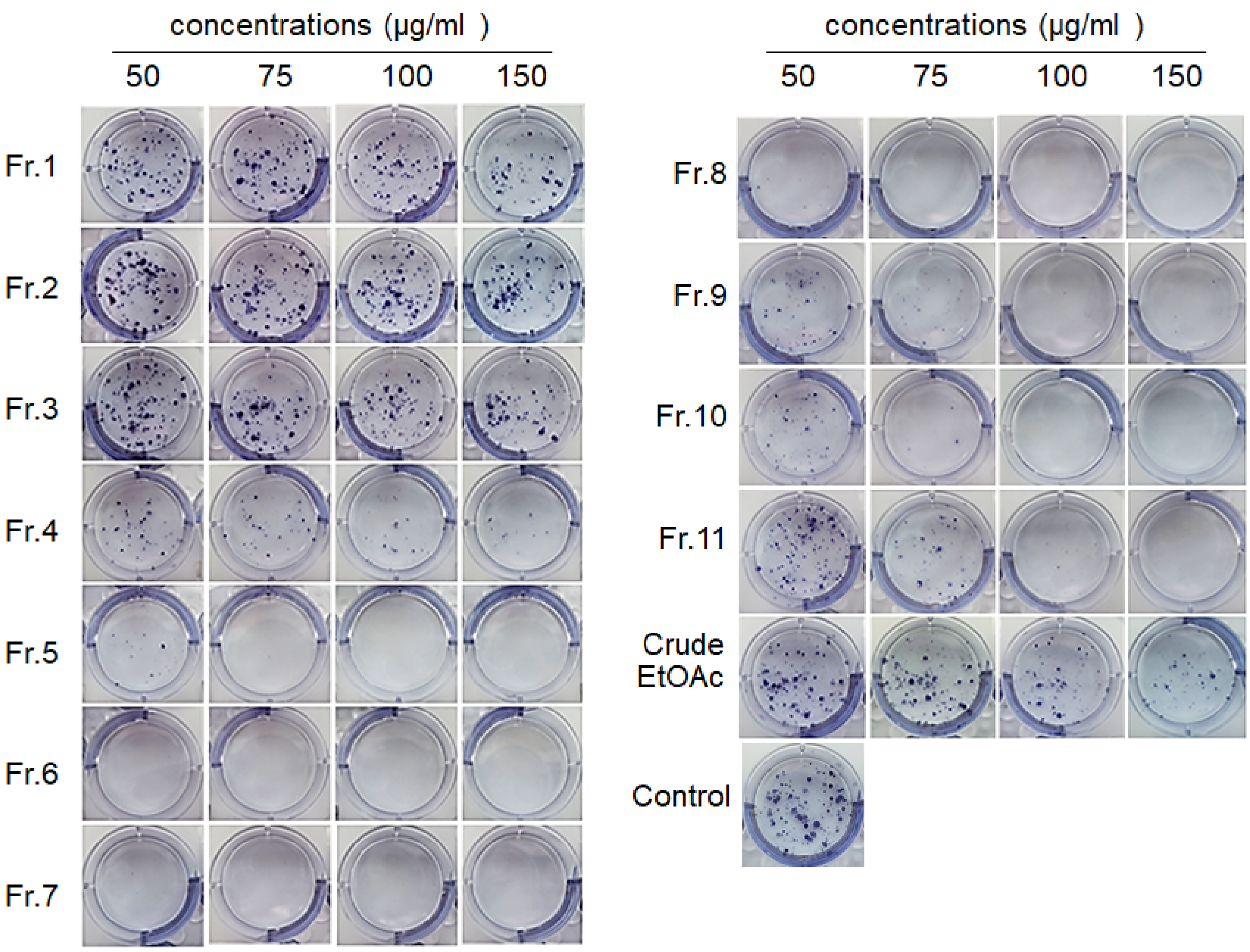

Supplementary Figure S1. Colony formation assay. MDA-MB-231 cells were treated with crude EtOAc extract and its fractions at concentration 50 – 150 μg/ml for 24 h. Cells were then cultured for 14 days in complete medium to determine ability of a single cell to grow into a colony. EtOAc, ethyl acetate; Fr, fraction.

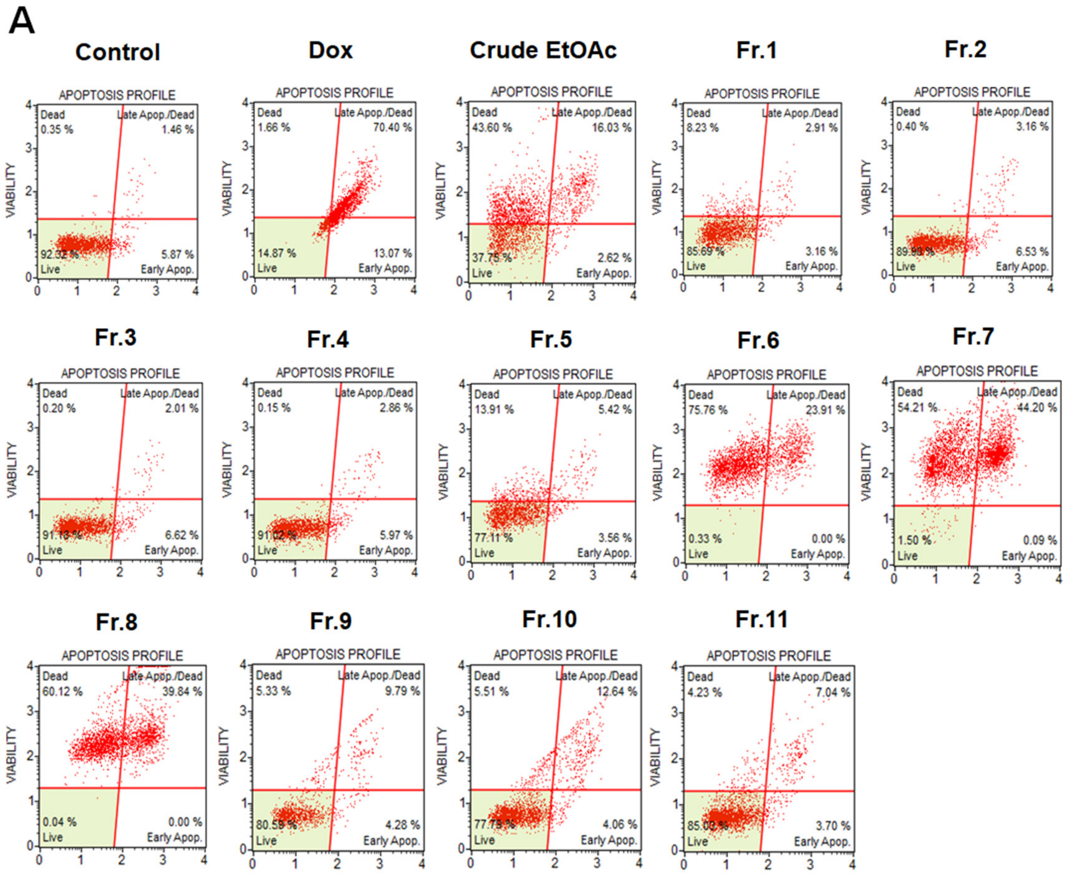

Supplementary Figure S2. Induction of apoptosis in MDA-MB-231 cells. Cells were incubated with crude EtOAc extract, 11 fractions (150 µg/ml) or doxorubicin (1.5 µM) for 24 h. For control, cells were incubated with complete medium alone. The upper-left quadrant (annexin V–, 7-AAD +) represent dead cells. The lower left quadrant (annexin V–, 7-AAD –) represent live cells. One-way ANOVA was performed with multiple comparison correction (Dunnett test). EtOAc, ethyl acetate; Fr, fraction; Dox, doxorubicin; 7-AAD, 7-amino-actinomycin D.

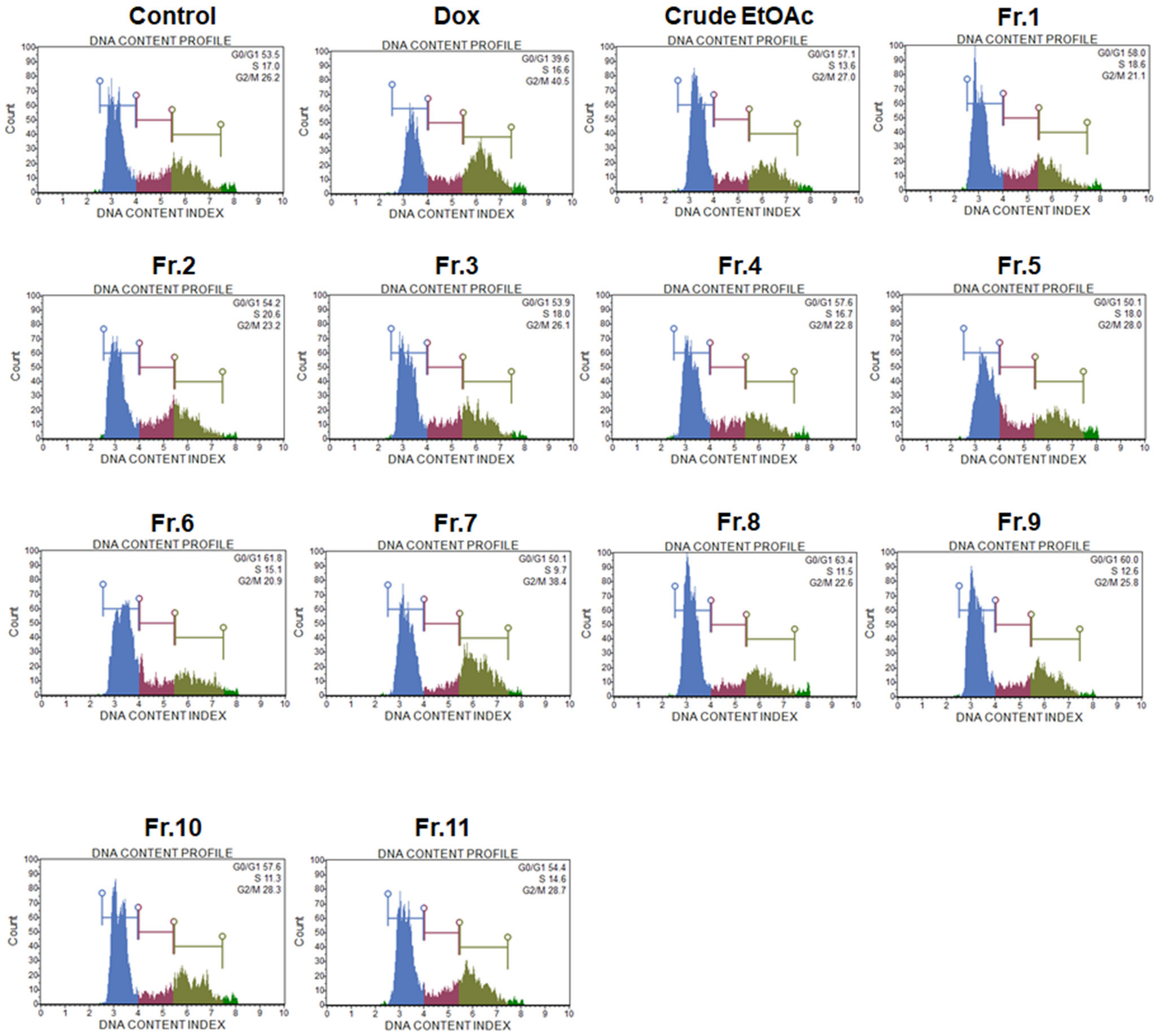

Supplementary Figure S3. Effect of MOL extract and its derived fractions on the distribution of MDA-MB-231 cells in the cell cycle. Cells were incubated with crude EtOAc extract, 11 fractions (150 µg/ml) or doxorubicin (1.5 µM) for 24 h. For control, cells were incubated with complete medium alone. EtOAc, ethyl acetate; Fr, fraction; Dox, doxorubicin.

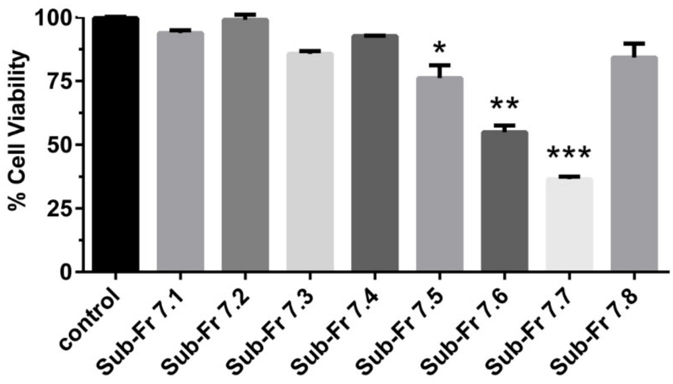

Supplementary Figure S4. Cell viability of MDA-MB-231 cells after treatment with sub-fractions no.7.1-7.8. Cells were incubated for 24 h with 75 µg/ml of each sub-fraction. One-way ANOVA test was performed with multiple comparison corrections (Dunnett test). Data represent mean ± SEM of three independent experiments. (*$p < 0.05$, **$p < 0.01$, ***$p < 0.001$). Fr, fraction

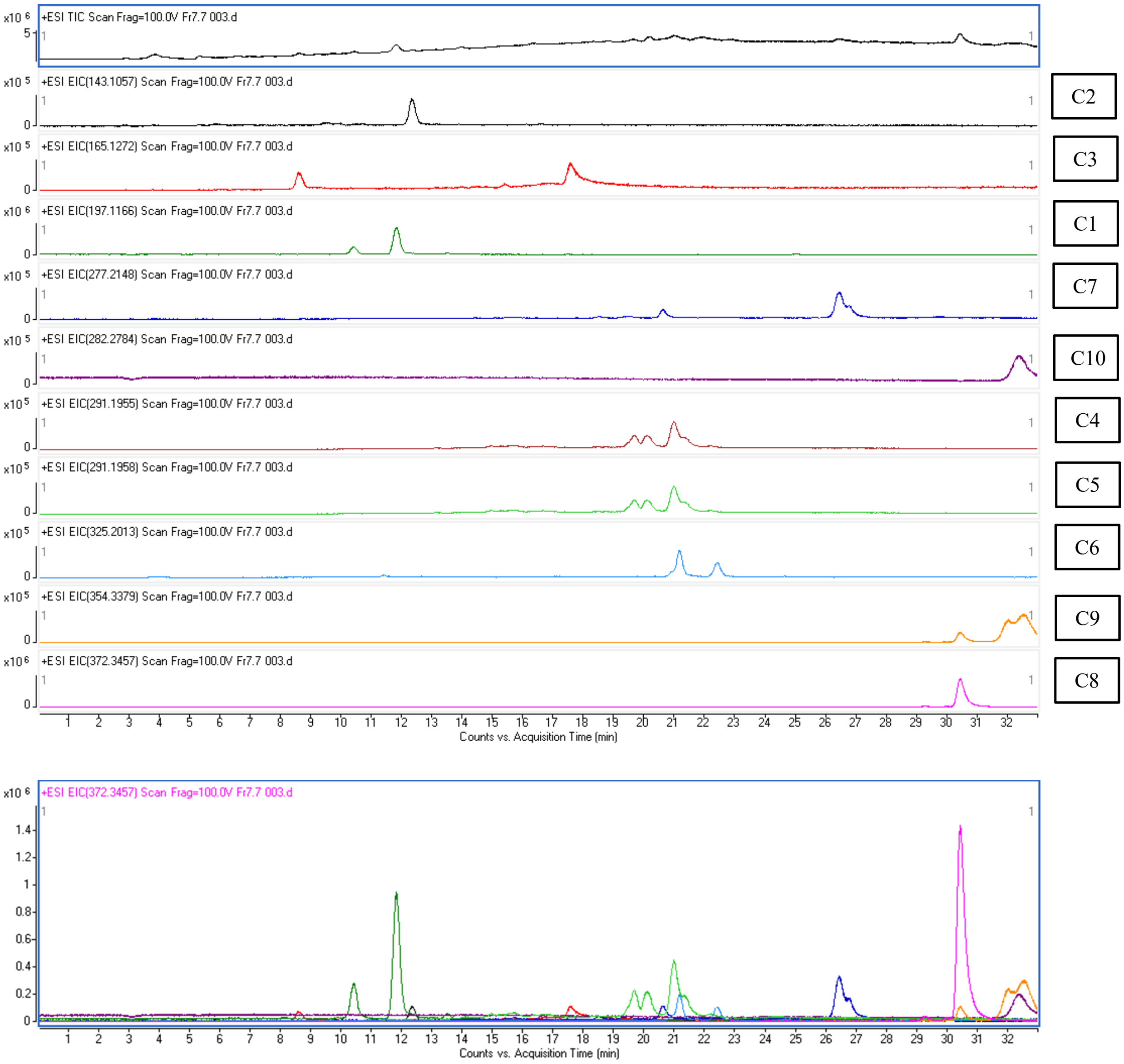

Supplementary Figure S5. Overlays of LC-MS chromatograms of MO sub-fraction no.7.7 with active compounds no.1-10.

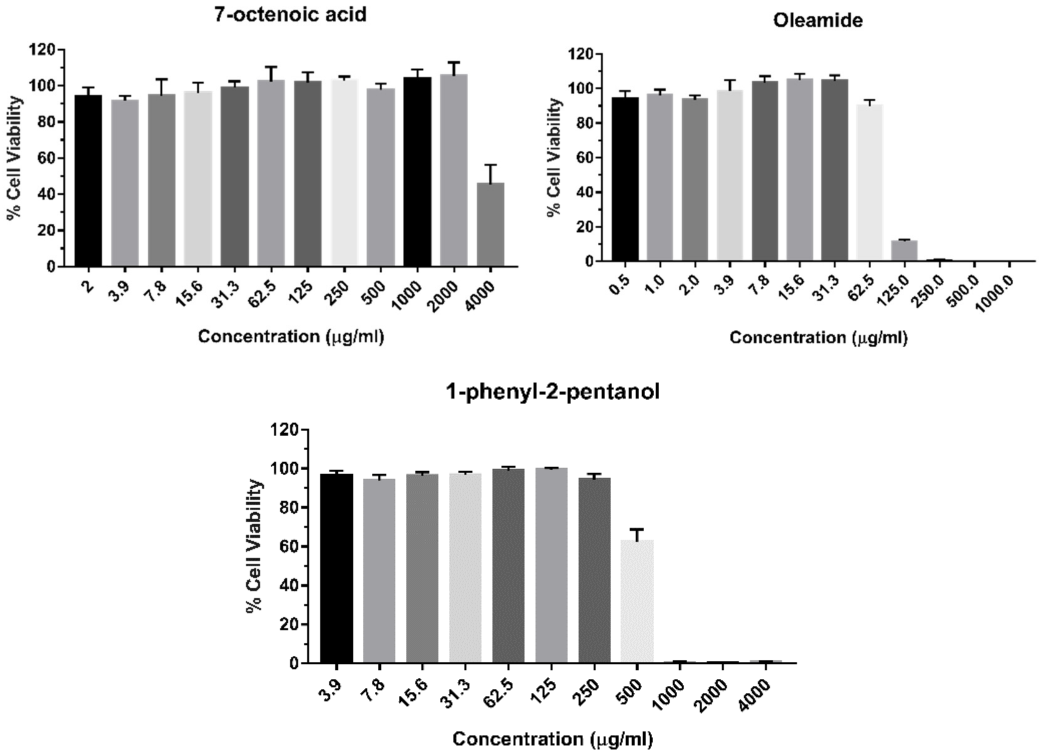

Supplementary Figure S6. Cytotoxicity of identified compounds on MDA-MB-231 cells. Cells were plated on 96-well plates and incubated with increasing concentrations of 7-octenoic acid, oleamide, and 1-phenyl-2-pentanol for 24 h. The viability was measured by using MTT assay. Each bar graph represents mean + SEM.

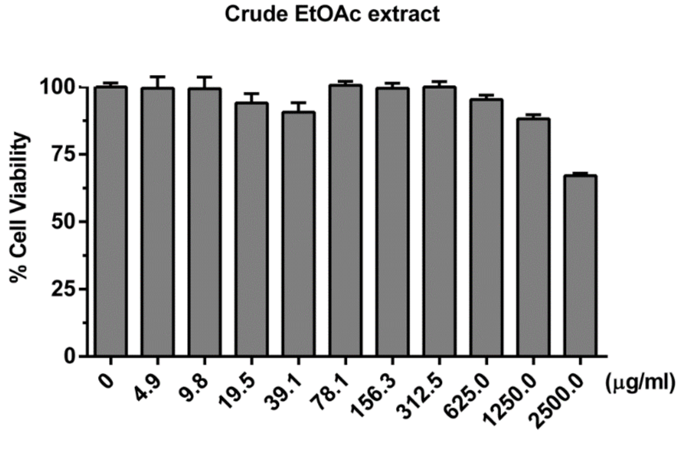

Supplementary Figure S7. Cell viability of primary human macrophage after treatment with crude EtOAc extract. Human macrophages were plated into 96 wells and incubated with increasing concentrations (0-2.5 mg/ml) of extract for 24 h. Cells viability was assessed by MTT assay. Data represent mean ± SEM of three independent experiments.

A

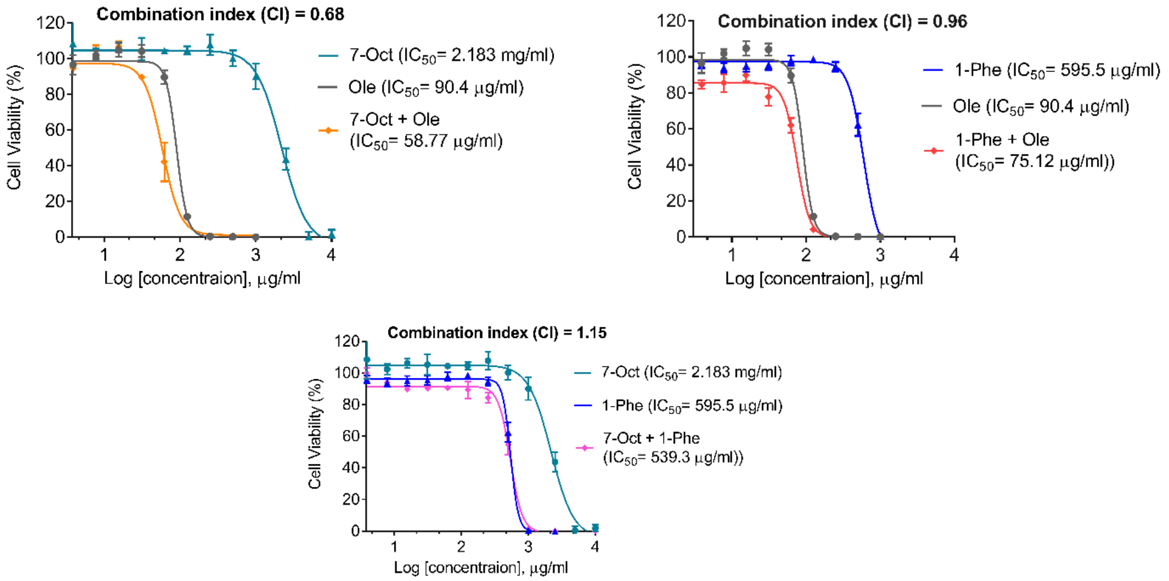
Combination index (CI) = 0.68
Cell Viability (%)
Log [concentraion], µg/ml
7-Oct (IC50= 2.183 mg/ml)
Ole (IC50= 90.4 µg/ml)
7-Oct + Ole (IC50= 58.77 µg/ml)
Combination index (CI) = 0.96
1-Phe (IC50= 595.5 µg/ml)
Ole (IC50= 90.4 µg/ml)
1-Phe + Ole (IC50= 75.12 µg/ml))
Combination index (CI) = 1.15
7-Oct (IC50= 2.183 mg/ml)
1-Phe (IC50= 595.5 µg/ml)
7-Oct + 1-Phe (IC50= 539.3 µg/ml))

B

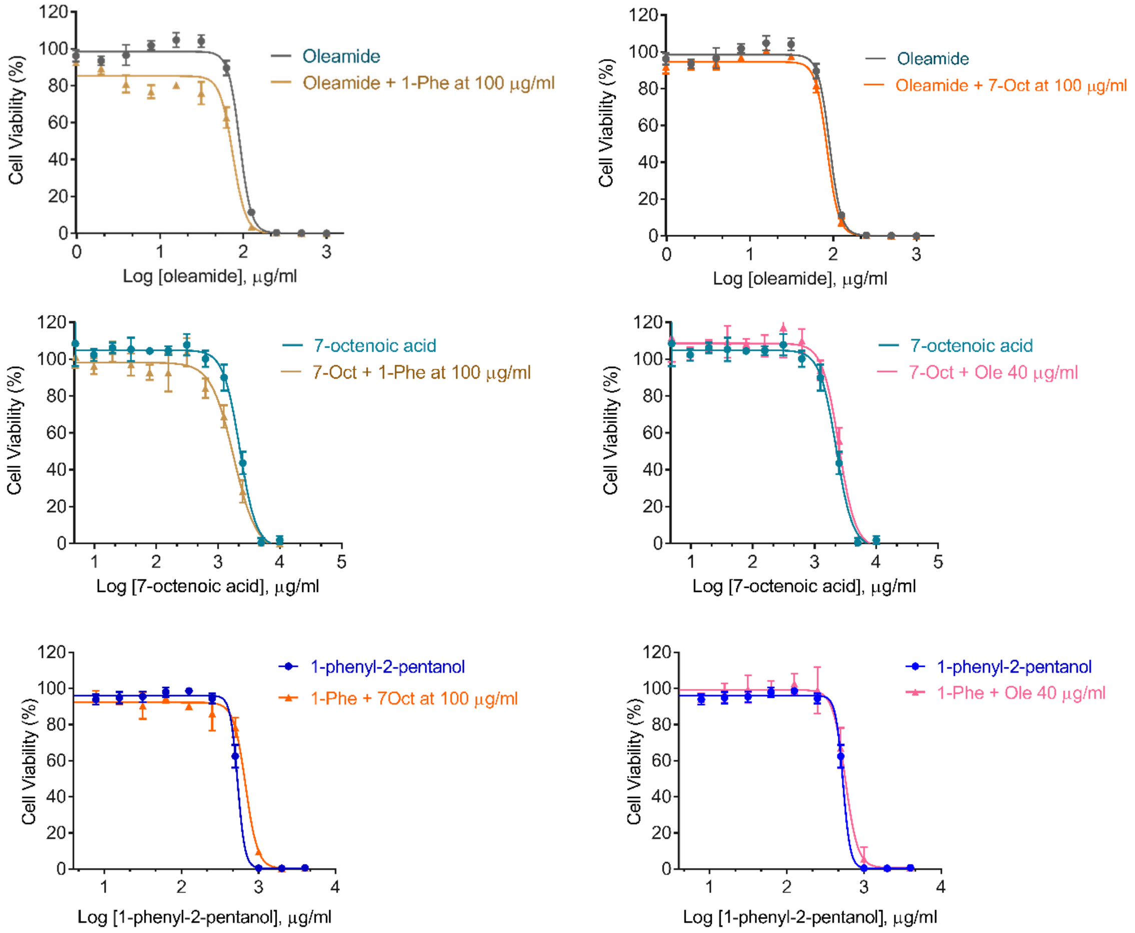
Cell Viability (%)
Oleamide
Oleamide + 1-Phe at 100 µg/ml
Log [oleamide], µg/ml
Oleamide
Oleamide + 7-Oct at 100 µg/ml
Log [oleamide], µg/ml
7-octenoic acid
7-Oct + 1-Phe at 100 µg/ml
Log [7-octenoic acid], µg/ml
7-octenoic acid
7-Oct + Ole 40 µg/ml
Log [7-octenoic acid], µg/ml
1-phenyl-2-pentanol
1-Phe + 7Oct at 100 µg/ml
Log [1-phenyl-2-pentanol], µg/ml
1-phenyl-2-pentanol
1-Phe + Ole 40 µg/ml
Log [1-phenyl-2-pentanol], µg/ml

Supplementary Figure S8. Combination effect of compounds on MDA-MB-231 cell viability. (A) Cells were incubated with increasing concentration of compounds alone (7-Oct, Ole, and 1-Phe) or compound combinations (7-Oct + Ole, 1-Phe + Ole, and 7-Oct + 1-Phe). (B) Cells were incubated with increasing concentration of compounds alone or combined with 1-Phe (100 µg/ml), 7-Oct (100 µg/ml), or oleamide (40 µg/ml). Cells were incubated for 24 h in all experiment. The viability was measured by using MTT assay. Each bar graph represents mean $\pm$ SEM of three independent experiments. 7-Oct, 7-octenoic acid; 1-Phe, 1-Phenyl-2-pentanol, Ole, oleamide. The combination index (CI) was calculated based on the IC_{50} values obtained from the MTT assay by using the formula;

$$CI = [IC_{50}(A+B)/ IC_{50}(A)] + [IC_{50}(A+B)/ IC_{50}(B)]$$

where IC_{50} (A) and IC_{50} (B) are the IC_{50} values obtained from each compound separately. IC_{50} (A + B) is the IC_{50} value of both compounds in combination.

Beta-actin (42 kDa)

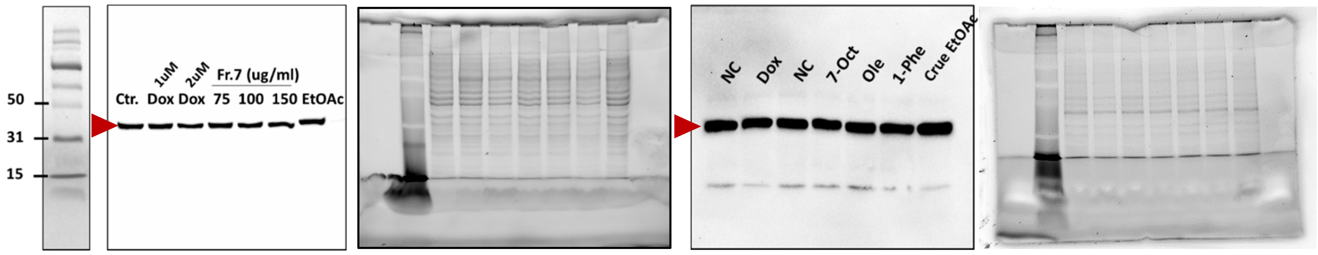

Bcl-2 (26 kDa)

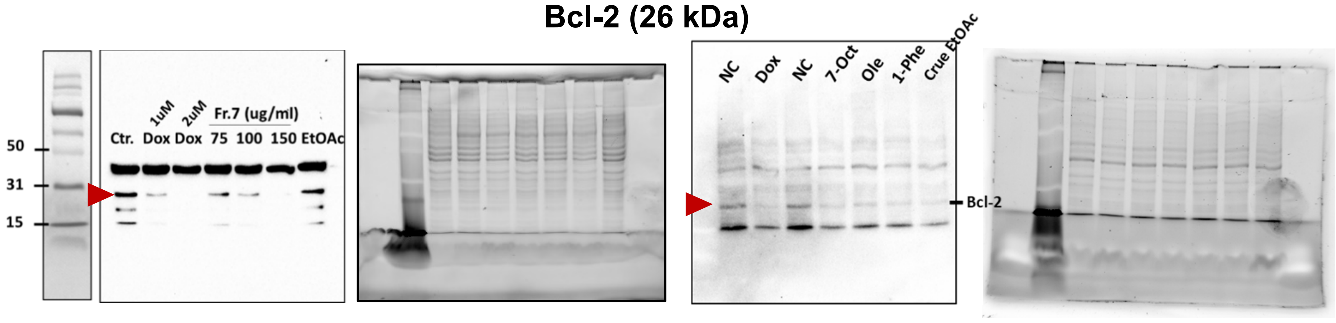

Bax (23 kDa)

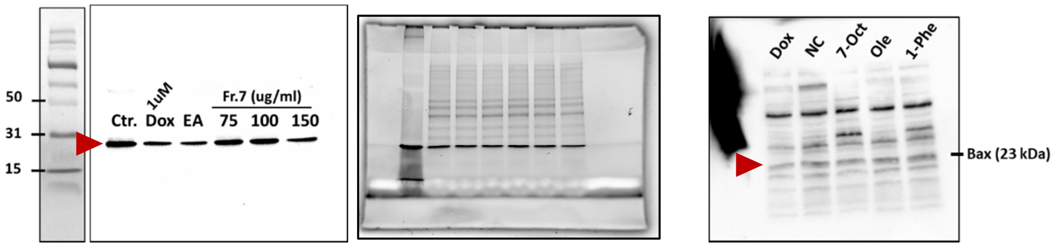

Pro-caspase 3 (32 kDa)

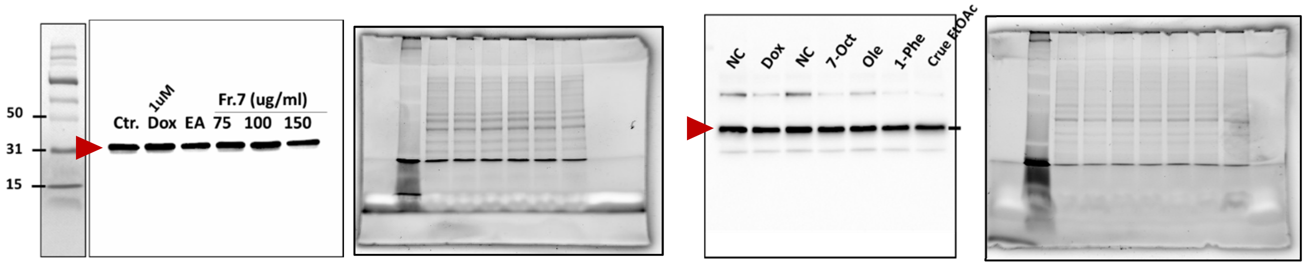

Cleaved caspase 3 (17 kDa)

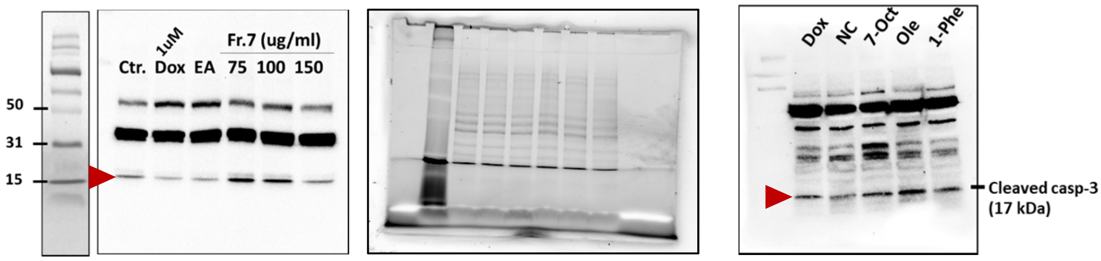

Supplementary Figure S9. Original Images of Blots and Gels. MDA-MB-231 cells were incubated with fraction no.7 or compounds for 24 h. The membranes were probed with Beta-actin, Bcl-2, Bax, pro-caspase 3, and cleaved caspase-3, respectively.
